# Publisher Correction:

**DOI:** 10.1038/s44276-024-00040-2

**Published:** 2024-04-05

The article metadata for the following articles initially included an error in the publication year: 10.1038/s44276-023-00001-1, 10.1038/s44276-023-00002-0, 10.1038/s44276-023-00003-z, and 10.1038/s44276-023-00004-y. This has since been corrected to 2023.

Additionally, the article citation for 10.1038/s44276-023-00006-w has now been updated as its previous article numbering overlapped with that of another article.

